# Micro-Hole Generation by High-Energy Pulsed Bessel Beams in Different Transparent Materials

**DOI:** 10.3390/mi12040455

**Published:** 2021-04-18

**Authors:** Valeria V. Belloni, Monica Bollani, Shane M. Eaton, Paolo Di Trapani, Ottavia Jedrkiewicz

**Affiliations:** 1 Dipartimento di Scienza e Alta Tecnologia, Università degli Studi dell’Insubria, Via Valleggio 11, 22100 Como, Italy; valeria.belloni@femto-st.fr (V.V.B.); paolo.ditrapani@uninsubria.it (P.D.T.); 2 FEMTO-ST Institute, University Bourgogne Franche-Comté, UMR CNRS 6174, 15B Avenue des Montboucons, 25030 Besançon, France; 3 Istituto di Fotonica e Nanotecnologie (IFN)—CNR, L-NESS, Via Anzani 42, 22100 Como, Italy; monica.bollani@ifn.cnr.it; 4 Istituto di Fotonica e Nanotecnologie (IFN)—CNR, Piazza Leonardo da Vinci 32, 20133 Milano, Italy; shane.eaton@ifn.cnr.it; 5 Istituto di Fotonica e Nanotecnologie (IFN)—CNR, Udr di Como, Via Valleggio 11, 22100 Como, Italy

**Keywords:** laser fabrication, micro-hole drilling, pulsed Bessel beams, transparent materials

## Abstract

Micro-drilling transparent dielectric materials by using non-diffracting beams impinging orthogonally to the sample can be performed without scanning the beam position along the sample thickness. In this work, the laser micromachining process, based on the combination of picosecond pulsed Bessel beams with the trepanning technique, is applied to different transparent materials. We show the possibility to create through-apertures with diameter on the order of tens of micrometers, on dielectric samples with different thermal and mechanical characteristics as well as different thicknesses ranging from two hundred to five hundred micrometers. Advantages and drawbacks of the application of this technique to different materials such as glass, polymer, or diamond are highlighted by analyzing the features, the morphology, and the aspect-ratio of the through-holes generated. Alternative Bessel beam drilling configurations, and the possibility of optimization of the quality of the aperture at the output sample/air interface is also discussed in the case of glass.

## 1. Introduction

Micro-hole drilling of transparent materials plays an important role in the field of micromechanics, microelectronics, microbiology, and microphotonics [1]. Photolithography is often applied to facilitate micro-drilling, but it requires advanced facilities and a high number of processing steps, and is limited in material type and geometry. On the other hand, lasers are now widely applied in scientific and industrial applications, offering highly directional and localized radiation facilitating material modifications at precise locations [2]. Ultrafast pulsed laser systems are especially important for the growing industrial demand for fast processing and for micrometer scale high-quality devices [3], overcoming the limitations due to the production of cracks or low fabrication rates of conventional methods such as diamond drilling, water jet drilling and micro-sand blasting [4]. Moreover, the use of ultrashort pulses such as femtosecond or picosecond pulses allows the deposition of energy into the material before thermal diffusion occurs, resulting in the reduction of heat-affected zones and, as a result, more precise and smoother features [5].

The laser beam shape and the beam propagation features also play a crucial role in determining the hole quality, and research continues to be carried out for the optimization of the system performance such as speed, throughput, sidewall taper, or symmetry [6,7,8]. In particular, an important advance in ultrafast laser processing has been through the development of “non-diffracting” beams, which enable overcoming many of the difficulties usually encountered with standard Gaussian beam focusing in materials [9,10]. Finite energy Bessel beams (BB) [11], for instance, can offer advantages when drilling micron-sized holes, thanks to their cylindrical symmetry and their cross-section featured by an intense central spot surrounded by weaker concentric rings, these being the energy reservoir during propagation. Thanks to their self-reconstruction property and their elongated focal zone, BB allow an easier manipulation of the diameter and depth of the microchannels [12,13,14] or micro-holes to be created [15,16,17]. In general, Bessel beams have remarkable characteristics and can be used for high impact applications [18] such as high-speed cutting [19,20] and cleaving [21,22], or welding [23] of transparent materials.

The laser micro-drilling process applied in this work is based on the combination of machining with such non-diffracting beams with a trepanning-like technique, already used for the etching of glass–air interfaces and for the generation of through-holes in thin glass samples [24,25]. Here, the experimental investigation has been carried out on diverse types of glass, poly(methyl metacrylate) polymer (PMMA), and synthetic diamond. All the chosen materials are transparent in the near-infrared, but exhibit different behaviors during the light–matter interaction due to their varied optical, thermal, and mechanical properties. The aim of this work is to show that this BB micro-drilling technique already tested in thin glasses can be extended to thicker materials, and can be performed as in the previous case without scanning the beam position along the sample thickness. We highlight the cases where the different material properties prevent from reaching high quality results, providing a starting point for future deeper investigations about radiation–matter interaction with Bessel beams. We first compare the results obtained for through-holes, with diameter on the order of tens of micrometers, generated in glass bulk samples with different thicknesses. In particular for the AF32 glass, we also present alternative drilling configurations, with some results of BB drilling in water, and we discuss the possibility of optimization of the quality of the aperture at the output sample/air interface, by using an adhesive layer on the bottom surface of the sample. For PMMA, we first present results related to microstructures generated in the bulk for single- and multiple-shot Bessel beam machining, in order to highlight the differences with respect to previous investigations in glass [24], and we illustrate the drawbacks of the BB laser microfabrication in this material. We highlight the successful outcome of the micro-drilling of a 500 μm-thick synthetic diamond. In all cases, we present optical microscope images of the machining outcome, and we discuss the morphology and quality of the void structures obtained, especially at the air/sample interfaces, where the features of the apertures strongly depend not only on the material characteristics but also on the laser beam parameters.

## 2. Materials and Methods

In this work, we tested four different types of transparent materials: three different borosilicate glasses (AF32 and D263 from Schott, Mainz, Germany, and Eagle XG from *Corning,* New York, NY, USA), PMMA, and diamond. The thermal and mechanical characteristics of the materials are reported in Table 1. The glass samples have similar properties between them but different thicknesses, while the other two material samples have very different optical, thermal, and mechanical properties, and we expect significant differences in the outcome of the laser–matter interaction process.

The micromachining experiments have been performed by means of a 20-Hz Ti:Sapphire amplified laser system (Amplitude Technologies) delivering 40-fs transform-limited pulses at 800 nm wavelength in mJ range pulse energy. This laser source allows us to have enough energy distributed along the BB non-diffracting length at the sample position, in order to guarantee a strong modification in a single shot along the whole bulk thickness, a necessary condition to drill apertures in dielectric materials [24]. By detuning the laser compressor, the pulse can be stretched and the pulse duration is adjusted in the picosecond range for an efficient high aspect-ratio bulk modification and a confined energy deposition (nonlinear absorption) in the regime of single shot BB machining [14,24].

It is worth noting that, during the radiation–matter interaction, different pulse widths influence the nonlinear absorption process and the laser machining outcome of transparent materials. The pulse duration is indeed an important control parameter, since the energy deposition mechanism can be different in the case of long or short pulses [32], and the deposited energy distribution is directly proportional to the pulse duration [33]. Moreover, the physical phenomena associated with the interaction of an ultrashort laser pulse with transparent materials occur on different time scales. In particular, the carrier excitation due to the absorption of photons and to the avalanche ionization occurs in the femtosecond and picosecond time scale, respectively, followed by thermalization and then structural events (in a time scale going from tens of picoseconds to nanoseconds, and up to millisecond for resolidification). Contrarily to what happens with long pulses (e.g., ns pulses), where the energy is deposited in the material by the laser pulse and is transported out of the irradiated region by thermal diffusion, the use of ultrashort pulse durations in the fs range allows a controlled and localized delivery of thermal energy during the nonlinear absorption process [5]. Our choice to work in the picosecond regime derives from the fact that, in the case of laser micromachining with Bessel beams, optimal energy and pulse duration parameters must be chosen in order to generate in single shot uniform elongated microstructures in transparent materials, especially in the case of thick samples [34]. In particular, picosecond pulses take advantage of nonlinear plasma absorption and avoid temporal dynamics effects which can compromise the stationarity of the Bessel beam propagation, the latter corresponding to the requirement that the Bessel beam refilling length should be smaller than the nonlinear length of the material [14,35], a regime identified as dominated by nonlinear losses in [36].

**Figure 1:**
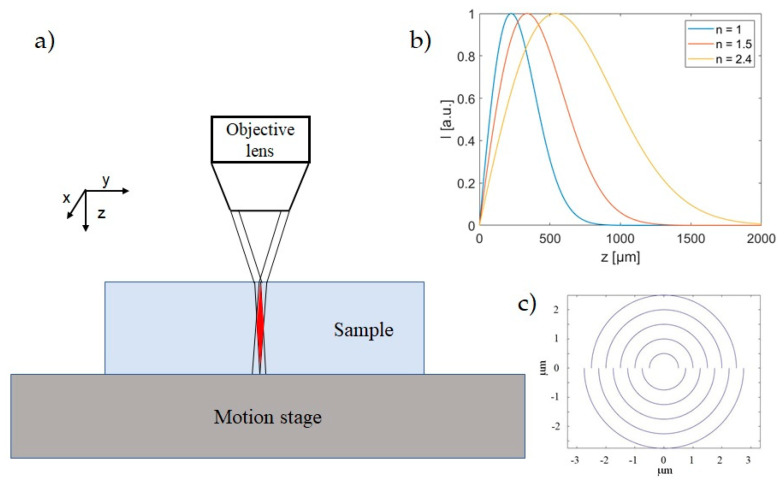
(**a**) Scheme of the relative positioning of the Bessel beam with respect to the sample (the Bessel zone is shown in red) for an optimal energy deposition along the whole sample thickness during the laser machining [18]. Note that here, just for illustration, the Bessel zone is arbitrarily represented with a length equal to the sample thickness; (**b**) normalized intensity profiles of a Bessel beam with cone angle 12° calculated in three different media (air, borosilicate glass and diamond respectively); (**c**) schematic example of spiraling trajectories programmed with SCA for the hole drilling.

The experimental set-up is similar to that illustrated in [25]: the spatially filtered Gaussian laser beam (5 mm size beam waist) is incident on a fused silica axicon with base angle 1°, and generates a BB which is demagnified by a suitable telescopic system constituted by a lens (focal length *f*_1_ = 250 mm or 300 mm) and a 0.45 N.A. 20× microscope objective (*f*_obj_ = 9 mm). To deflect the laser beam and send it vertically towards the sample placed on a micrometer precision motorized stage (driven by SCA, system control application software, *Altechna Rnd,* Vilnius, Lithuania), a dichroic mirror is positioned between the lens and the objective. The resulting Bessel beam impinging orthogonally onto the sample (Figure 1a) is characterized in air before starting the micromachining process by means of an imaging system. Depending on the chosen focal length *f*_1_, this BB is featured by a cone angle *θ* of 12° or 15°, corresponding to a central core size in the range of 1.2–1.4 μm and a total Bessel zone (non-diffractive length) in air of about 820 μm or 570 μm, respectively. During the laser writing, a real-time imaging of the back-illuminated sample surfaces is also performed onto a CCD camera. This also allows a careful adjustment of the relative positioning of the BB focal length (and thus of the core intensity profile along the propagation direction) with respect to the sample [25]. Note that this length increases by a factor equal to the refractive index of the transparent medium (see, for illustration, Figure 1b). The adjustment of the beam position allows us to optimize the beam interaction along the whole sample thickness. In particular, the BB is positioned in a such a way that the Bessel zone is symmetrically distributed across the sample, i.e., with maximal intensity in the middle of the sample. It is worth noting that the propagation inside a material with higher refraction index than air causes a reduction of the beam cone angle in the sample and an elongation of the Bessel zone (as it will be described in Section 3.1.4). Therefore, the pulse energy of the BB is spread over a longer focal distance causing a reduction of the local beam intensity along the propagation direction in the medium.

During the BB drilling process, the writing trajectories are spiraling-like trajectories (see Figure 1c) starting from the center of the zone to be machined as described in [25]. In the work reported in [25], a study on the different parameters affecting the hole quality and morphology, such as the distance between the circles (or more precisely semi-circles) used to draw the spiral (the so-called “step”), the number of circles, the density of pulses (pulses mm^−1^), and the speed of fabrication, was performed. While the step parameter affects the radial density of the impinging pulses, changing the number of circles modifies the size of the holes, with a minimum needed number (for a maximum aspect ratio of the hole); this number also depends on the thickness of the sample to drill. It is worth noting that, for a given repetition rate of the laser system, the fabrication speed determines the pulse density simply according to:

Pulse density = LRR/speed = 1/(speed × Δ*t*),

where LRR is the laser repetition rate and Δ*t* the temporal separation between the single pulses. The pulse density and the pulse energy must be carefully adjusted in order to induce a suitable damage inside the bulk along the BB interaction zone associated with each pulse, and to guarantee a partial superposition of neighboring BB focal lines (i.e., interaction zones in the material) [24].

## 3. Results

The laser micromachining of the various transparent materials has been performed by using the two beam geometries described in the previous section, depending on the thickness of the sample to drill. We have chosen the BB with shortest Bessel zone (cone angle 15°) for the AF32 glass sample, and the BB with longer Bessel zone (cone angle 12°) for the other thicker material samples. Note that BB micro-drilling tests of thin borosilicate and AF32 glass were previously achieved by our group with a high repetition rate laser source [24,25]. In particular, for the AF32 material, the dependence on the number of circular writing trajectories and on the fabrication speed was investigated in the single pulse mode machining regime in the 2–30 kHz range; in that context, a comparison with the burst mode regime was also explored. In contrast to those previous works, here we take advantage of the high power 20 Hz Ti:Sapphire laser working in a single pulse regime, and we repeat the drilling process in order to be able to compare the results obtainable with thicker and different materials, for which more energy is needed. Indeed, the important point is the possibility to have high energy BB pulses at the sample, so here we do not focus on the speed of fabrication but simply on the possibility to achieve as a proof-of-principle the through-micro-holes in the different dielectrics. We should also note that, in terms of the thermal processes that may be involved in the laser–matter interaction, because of the low repetition rate, the present fabrication regime cannot be considered the same as that when using a high repetition rate laser, so a comparison with previous results [18] will not be considered.

We point out that, for this work, machining tests with different pulse durations in the picosecond regime, and with different pulse energies, have been preliminarily carried out by injecting the BB in single shot through the dielectric samples. This is done typically close to a lateral edge in order to observe under a microscope the internal bulk modification and thus the deposition of energy along the whole thickness of the sample to drill. This preliminary study, together with the optimization of the laser writing parameters during the drilling process (i.e., pulse density, number of circular writing trajectories), has allowed us to assess the optimal conditions for the achievement of a through-hole in the different samples. The pulse duration was set to 6 ps apart from the PMMA case as discussed later on. For what concerns the writing parameters, we have found that, with the 20 Hz Ti:Sapphire laser source and our BB geometries, the highest fabrication speed that allows the drilling of a through-hole in glass is 0.01 mm s^−^^1^, corresponding to a pulse density of 2000 pulses mm^−^^1^, with a 0.25 μm step between the circular trajectories. We have verified that these parameters are suitable to drill PMMA and diamond as well and therefore they have been adopted in all our machining experiments.

### 3.1. Hole-Drilling in Glasses

#### 3.1.1. Schott AF32 Glass 

**Figure 2:**
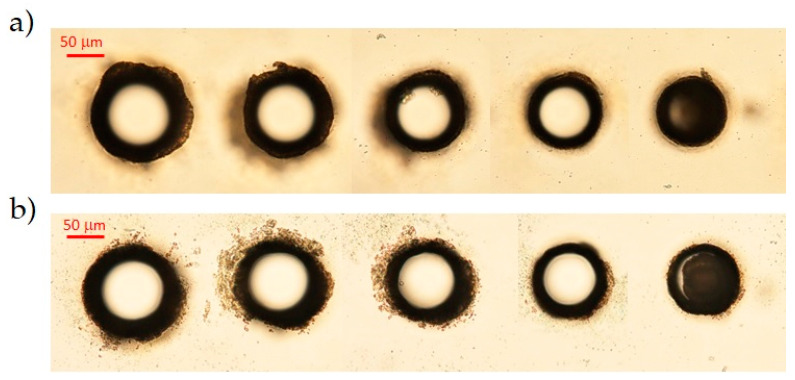
Optical microscope images (top (**a**) and bottom (**b**) surfaces) showing the dependence of the hole diameter on the BB pulse energy in the case of the 200 μm thick AF32 glass sample. From left to right, the energy per pulse was, respectively, 250 μJ, 197 μJ, 153 μJ, 118 μJ and 95 μJ (with 3% variance). The holes were drilled in one machining pass with 60 writing circles.

**Figure 3:**
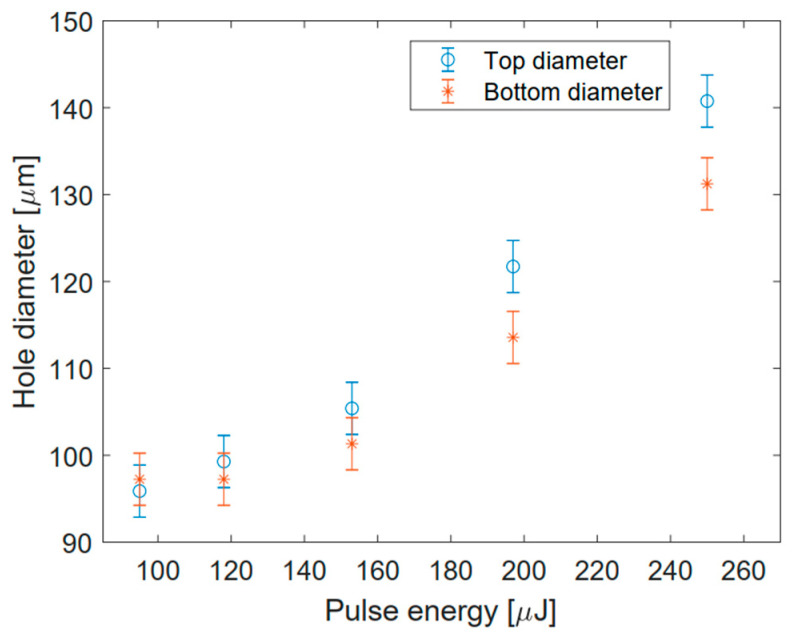
Dependence of the hole diameter on the BB pulse energy in the case of a 200 μm thick AF32 glass sample drilled in one machining pass with 60 writing circles. Energy and diameter values correspond to the images of Figure 2.

In order to extend previous results on the 200 μm-thick AF32 glass sample, we have started by studying different conditions to drill through-holes with different sizes in a low repetition rate regime. To this end, we have set to 60 the number of writing circles in the trepanning process, and we have checked the dependence of the hole diameter on the pulse energy. The results are presented in Figure 2, where we show top and bottom microscope images of five holes generated in one machining pass with pulse energies at the sample ranging, respectively, from 95 μJ to 250 μJ. Considering the apertures’ dimensions at the surfaces, it turns out that the diameter of the holes decreases with a decreasing energy pulse, as shown in the plot reported in Figure 3. This observation has then been generalized to the other glasses.

**Figure 4:**
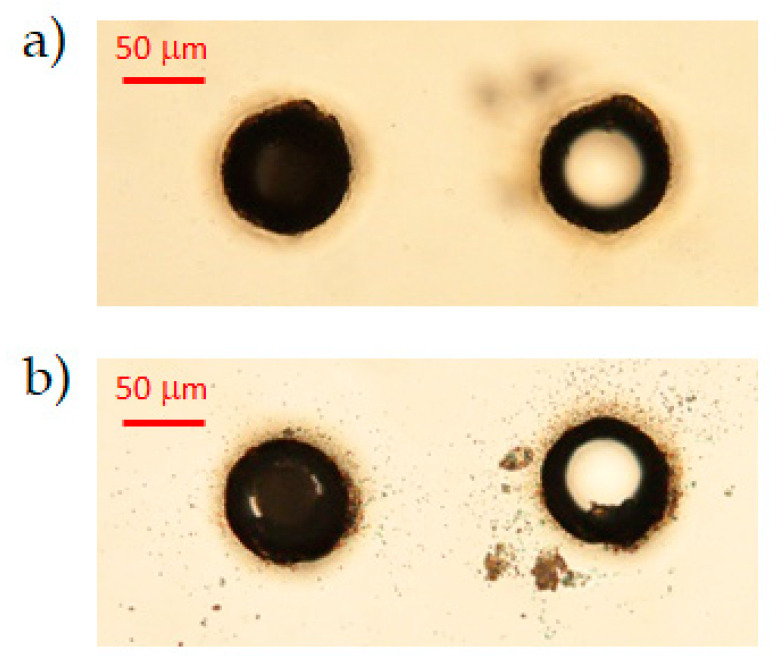
Optical microscope images of holes ((**a**) top surface, and (**b**) bottom surface) performed with 40 (left images) and 41 (right images) number of writing circles in the single pass BB micro-drilling process on a 200 μm thick AF32 glass sample (pulse energy 118 μJ).

For a pulse energy below 118 μJ, no through-hole could be drilled in the AF32 glass with the chosen fabrication parameters. On the other hand, the higher the amount of energy used, the more irregular the border of the apertures on the surfaces: it turns out that it is preferable to use a lower energy per pulse and to increase the number of writing circular trajectories in order to obtain more regular holes with a larger diameter. By fixing the pulse energy to 118 μJ, we have also found that the minimum number of writing circles needed to drill a through-hole in this 200 μm-thick sample is 41 (Figure 4). For given fabrication parameters, this value generally depends on the material thickness. By launching the BB drilling process twice (double pass machining in the same position), the threshold for the minimum number of circles required decreases to 35. Note that, in general, with this trepanning-like BB drilling technique, the minimum number of circles that can be used to generate a hole in the transparent material is also linked to the best aspect-ratio achievable. The latter is limited by the finite damage spot produced on the sample surfaces by the BB after each impinging shot. The superficial damage depends in fact both on the core beam dimensions and on the minimum number of spiraling trajectories needed to “dig” sufficiently the bulk before complete extrusion of the material from the central part [24].

**Figure 5:**
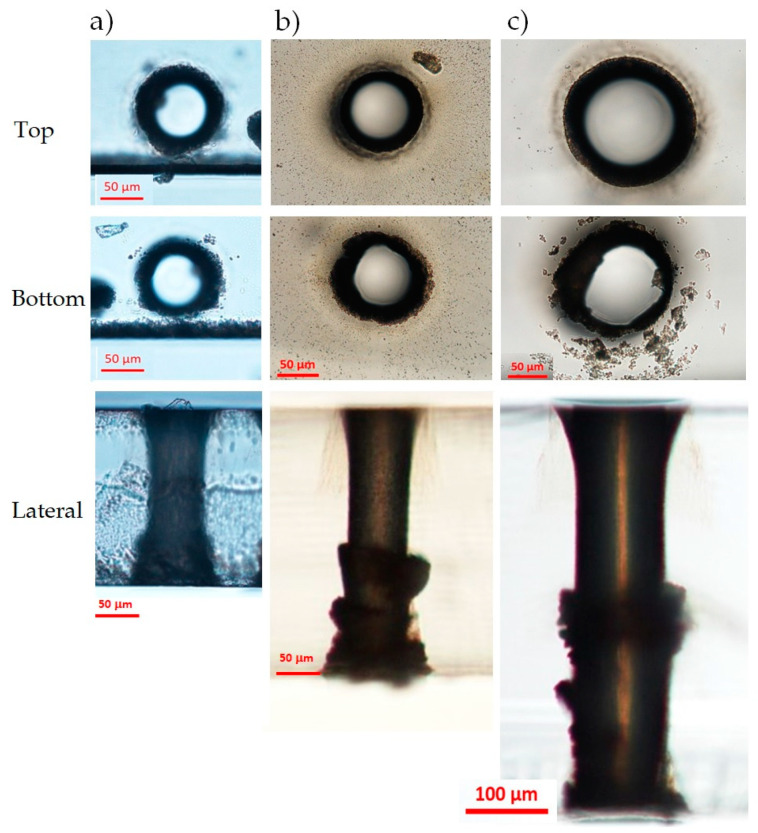
Optical microscope images (taken after an ultrasonic bath in acetone) of holes (top surfaces (first row), bottom surfaces (second row), lateral views (third row)) generated with the BB drilling technique in three different glass samples. (**a**) 200 μm thick AF32 glass (130 µJ pulse energy, 50 writing circles, one machining pass); (**b**) 300 μm thick D263 glass (300 µJ pulse energy, 55 writing circles, two machining passes); (**c**) 500 μm thick Eagle XG glass (390 µJ pulse energy, 100 writing circles, three machining passes).

In Figure 5a, we report microscope images (top, bottom, and lateral views, respectively in the first, second, and third rows) of a through-hole performed in single pass in the AF32 sample close to a lateral border, with a BB pulse energy of 130 μJ and 50 writing circles. The aspect-ratio of the hole is about 2.5. The entrance and exit apertures present a good circular shape, but, at the bottom surface, we can still observe some small irregularities at the border of the aperture. We attribute this to the refractive index change, which causes a sudden increase of the local beam peak intensity at the interface, as well as to the fact that the ablation threshold at the air/material interface is usually lower than the damage threshold inside the bulk [37]. Moreover, the bottom surface of the sample is the one that allows the expulsion of the ablated material during the micromachining process. Along the sample thickness, the hole shape is regular with a slight tapered shape. The internal surface is smooth, although, in this case, it presents a sort of explosion towards the bottom of the sample that may be due to the irregular lateral edge of the sample caused by a previous laser cutting. Note that, with the optimal writing parameters chosen and dictated by our 20 Hz repetition rate laser, the fabrication time for obtaining a through-hole was 8 min.

#### 3.1.2. Schott D263 Glass

The second sample we have tested is a D263 borosilicate glass with a thickness of 300 μm. The thermal and mechanical properties are comparable to those of the AF32 glass, so we can expect a similar outcome from the hole drilling process. The larger thickness has required a longer Bessel zone and therefore a higher pulse energy. Differently to the AF32 glass tests, here we have set a number of circles equal to 55 and the pulse energy to 300 μJ. To obtain a through-hole in this case, the number of fabrication passes must be two, since one single pass-machining is not sufficient to completely extrude the material inside the hole. In this case, the fabrication time was 17 min. The results are shown in Figure 5b. The top and the bottom surfaces show similar features to those obtained in the AF32 glass. The lateral view presents an explosion in the middle of the sample thickness and irregularities in the final part of the hole that could be due to the double-pass fabrication process. The width of the hole is about 100 μm, corresponding to an aspect ratio of 3:1. 

#### 3.1.3. Corning Eagle XG Glass

The Corning glass sample is a borosilicate glass with a thickness of 500 μm. Because of its thickness, consistently larger than that of the samples used so far, we performed preliminary fabrication tests in a single shot with different pulse durations (in the picosecond regime [26]) and with a pulse energy larger than 300 μJ; we have verified that the chosen pulse duration of 6 ps leads to an energy deposition along the whole thickness of the sample. 

We have performed a through-hole in the Corning sample by using a pulse energy of 390 μJ (the upper limit value in our set-up) and threemachining passes, without changing the other parameters. The fabrication outcome is shown in Figure 5c. The generated hole, with aspect ratio of nearly 5:1, presents a regular aperture at the top surface with a diameter of about 175 μm, but an irregular shape at the bottom surface. Note that, just after the machining, the internal part of the exit aperture at the material/air interface was partially filled with glass material residues that have been removed only after an ultrasonic bath in acetone. We believe that, in order to obtain a more regular exit aperture, a higher pulse energy or an additional fabrication pass would have been needed. Due to energy limitation and to the time-consuming process at a low repetition rate, we nevertheless did not try. The shape of the hole along the sample thickness is regular in the upper part, but presents some explosions in the middle, similarly to what was observed for the Schott D263 glass sample. Despite of its shape, the sharpness of the border of the exit aperture seems slightly more defined in the Corning glass sample compared to the Schott glass, probably because the local intensity of the BB at the glass–air interface is lower in the Corning material. The total time needed to drill a hole in the Eagle glass was 82 min. It is important to notice that, with a high repetition rate laser source (for instance delivering pulses at 20 kHz or higher), these results can be achieved within a few (or less) seconds.

#### 3.1.4. Alternative BB Drilling Configurations Applied to Af32 Glass: Optimization of the Hole Quality

While the top aperture of the through-hole generated with our BB drilling technique is usually featured by a neat contour and high quality, the bottom one always presents irregularities. One hypothesis is that the worse quality observed at the bottom surface may be due to the rise of the local intensity of the Bessel beam core in correspondence with the interface dielectric/air, caused by the sudden lowering of the refractive index [37]. It is also worth noting, as mentioned in Section 2, that, in general, the Bessel beam geometry changes during propagation across an interface. Indeed, from geometrical optics considerations, it can be shown by applying Snell law at the dielectric/air interface featured by two media with different refractive indices (for instance, n_bulk_ > 1 and n_air_ = 1), that the Bessel beam cone angle will undergo a modification. In the approximation of small angles, the relation between the beam cone angle θ in the material bulk (θ_bulk_) and that in the air (θ_air_) is given by

(1) $$\theta_{\mathrm{bulk}}≅\theta_{\mathrm{air}}/n_{bulk},$$

Showing that, in this case, the BB cone angle is reduced when the beam crosses the interface at the bottom of the sample. In addition, the Bessel beam focal depth and the core size will be accordingly modified. Recall that, for a given half-width $w_{0}$ of the Gaussian beam sent onto the axicon, the BB focal depth $z_{B}$ and the beam core size $r_{0}$ are respectively given by [12]:

(2) $$z_{B}≅w_{0}/tan\left(\theta \right)≅w_{0}/\theta$$

and

(3) $$r_{0}=2.405\;\lambda /2\pi \sin\left(\theta \right)$$

where λ is the laser wavelength. When the beam propagates through the interface, we therefore have in first approximation:

(4) $$z_{B}^{bulk}≅z_{B}^{air}n_{bulk}$$

(5) $$r_{0}^{bulk}≅r_{0}^{air}n_{bulk}$$

This indicates that both the focal length (along which the total pulse energy is distributed) and the central core of the BB (the core being responsible for the material modification) undergo a sudden reduction, thus contributing to the local intensity increase at the bottom sample surface. 

**Figure 6:**
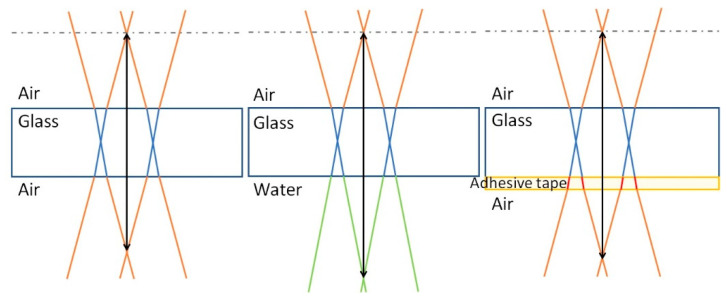
Schematic representation of the evolution of the Bessel beam geometry in different media. The intensity increase of the BB (core intensity) when propagating through the bottom surface is greater for the glass/air interface case (left) than for the glass/water interface case (center). On the other hand, the intensity of the BB decreases at the interface glass/adhesive tape.

Following these considerations, in the particular case of the AF32 glass sample, as a proof of principle investigation, we have therefore further applied the BB micro-drilling technique in conditions such to have a reduction of the refractive index change at the bottom interface of the sample with the external medium, and thus to have a lowering of the local intensity of the BB at that point—first, we have applied the BB drilling technique to a 200 μm-thick AF32 glass sample immersed in water, and second to a sample where a removable adhesive tape (*Kapton*) has been stuck to its bottom surface. The refractive indices and the theoretical cone angles of the Bessel beam in the media are reported in Table 2, and the modifications of the Bessel beam geometry during propagation through the various media are schematically represented in Figure 6.

(1) Micro-hole drilling in water

The liquid-assisted laser beam micromachining (LALBMM) is a microfabrication technique that uses liquids to improve the laser microfabrication of materials. The water is the most common liquid used because it is cheap, non-toxic, and transparent, and it can be combined with salts, bases, and organic additives to enhance its performance. In [38], many examples of improvements obtained with the LALBMM technique are reported with a wide range of laser parameters and materials. The features of the resulting backside etching of transparent materials obtained when focusing a Gaussian beam on the back surface of the sample are the etching threshold that is ten times lower than in gas, a low surface roughness in the fs-ps regime, and a small amount of debris.

**Figure 7:**
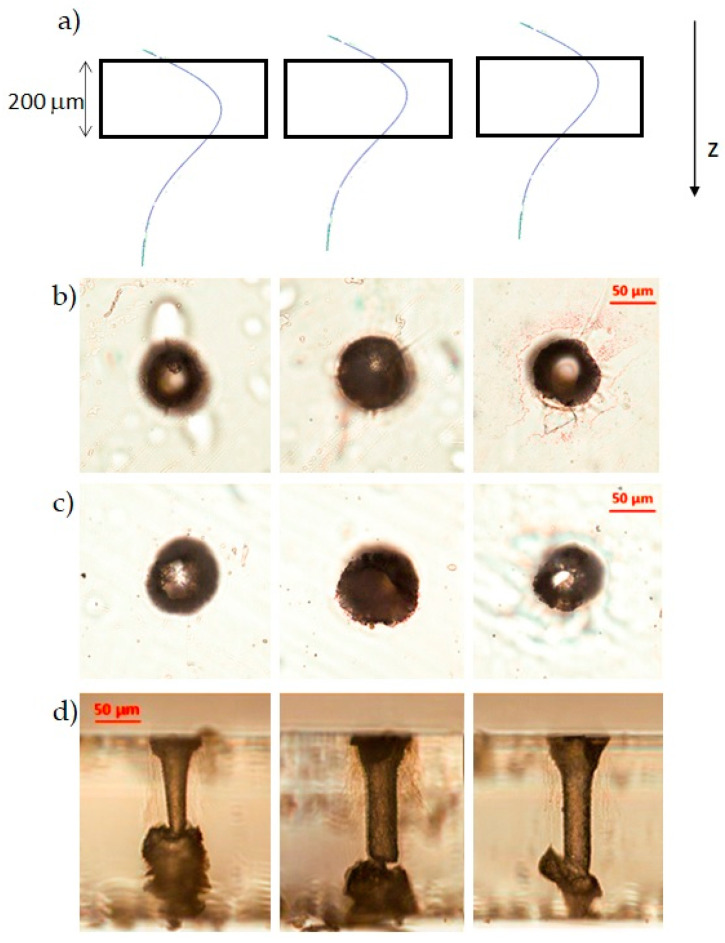
Optical microscope objective images of water assisted micromachining results in AF32 glass. (**a**) Schematic of the relative position between the BB (arbitrarily normalized) intensity profile along z and the sample thickness, in three different micro-drilling configurations; (**b**) top, (**c**) bottom, and (**d**) lateral views of the sample in the three configurations. In each column, the images correspond to a different sample position with respect to the Bessel zone of the beam. From right to left, the sample has been progressively shifted upwards towards the beginning of the Bessel beam intensity profile.

We have tried to use our BB micro-drilling technique together with the water assisted backside etching to see whether the results are different from those obtained in air. The 200 μm-thick AF32 glass sample has been fixed by two supports in a small cuvette filled with water, so that the water could only wet the bottom surface. The fabrication parameters used are the same as those reported in the previous section. The first tests have been performed in one machining pass, with a pulse energy of 132 μJ, 50 writing circles, and by, respectively, changing the relative position between the sample and the Bessel zone, i.e., shifting the sample along the *z*-axis. The results are shown in Figure 7a–d, where, in the microscope images shown from right to left, the sample has been progressively shifted towards the beginning of the Bessel beam. As illustrated in Figure 7a, only in the configuration of the second column was the intensity profile along the Bessel zone symmetrically distributed with respect to the sample thickness, while, in the machining configurations of the first and third columns, a relative shift between the Bessel beam and the sample thickness of 30 μm and 20 μm (respectively, and in opposite directions) was applied. All the channels generated present irregular shapes and cracks or explosions within the bulk along the sample thickness, the latter being maybe caused by the pressure wave generated in the water. We believe that the amount of damaged material due to the explosions in the vicinity of the bottom sample surface is greater (as in the first left image of Figure 7c) when the water interacts with a BB portion of increasing intensity. Moreover, during the real-time monitoring of the machining process, we could see that the online imaging of the sample got suddenly blurred after few tens of writing circles. Indeed, at the end of the laser fabrication, we have observed a thin film of water on the top surface of the sample and water on the microscope objective. Probably, the pressure produced at the bottom surface in the water during the laser–matter interaction facilitates the sudden generation of a through-hole. We believe that the water ejection and its deposition on the top surface certainly contributes to the Bessel beam distortion. However, the internal part of the hole shown in the microscope images in the right column of Figure 7 seems generally smooth. Surprisingly, it is featured by an internal diameter smaller than 40 μm, leading to an aspect-ratio of about 5:1, which is greater than that obtainable in air on the same material. This result has induced us to test the possibility of further reducing the internal diameter of the hole by using fewer writing circles (down to 30) during the trepanning process. We simply note that irregular through-holes very similar to those presented in Figure 7 were obtained (data not shown), but no conclusions could be drawn as the aperture dimension (unvaried) seemed to not be dependent on the number of circular writing trajectories. The non-deterministic and non-conclusive results of this experiment have been attributed to the presence of ejected water on the microscope objective.

**Figure 8:**
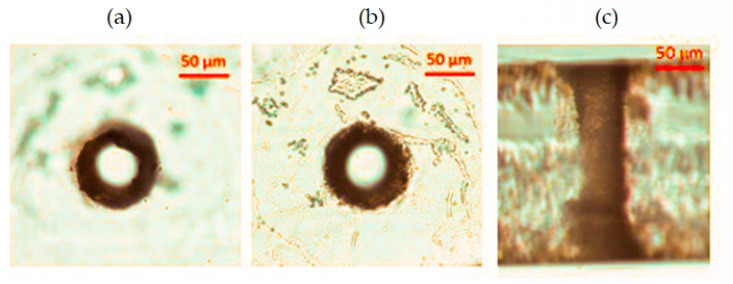
Optical microscope images of a through hole generated in a 200 μm-thick AF32 glass, in water assisted micromachining configuration, with writing trajectories starting from the aperture external contour towards its center, allowing for considerably reducing the fabrication time. Top (**a**), bottom (**b**) and lateral view (**c**).

A final drilling test with the sample immersed in water has been performed by using the BB trepanning technique in the opposite direction, which is by starting the writing trajectories from the external diameter of the wanted aperture till the center of the hole. In this case, the successful experiment has shown the possibility to drill a regular through-hole with an external diameter of 40 μm after only ten machining circles, therefore drastically reducing the fabrication time of the process (in our experimental conditions by a factor of about 3). The results are shown in Figure 8, where we observe that the apertures at the top and bottom surfaces present regular features, and the internal surface of the hole walls appears to be smooth. In contrast to Figure 5a, the internal hole diameter is in this case smaller than 50 μm. The aspect-ratio is about 1:4. We believe that bulk explosions such as those observed during the first BB drilling tests in water may play an important role in the complete extrusion of the material occurring in this machining configuration.

(2) Optimization of the bottom aperture quality by using adhesive tape

**Figure 9:**
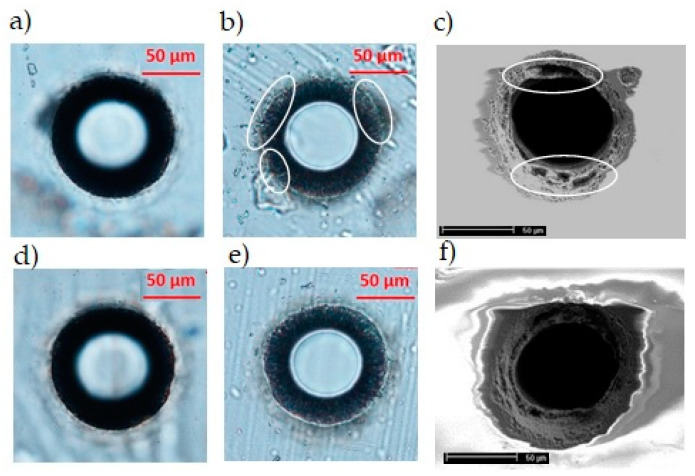
Through-holes generated in one pass and with a pulse energy of 130 μJ, in a 200 μm-thick AF32 glass, respectively without (top row) and with (bottom row), the adhesive layer of Kapton tape stuck on the bottom surface. (**a**,**d**) and (**b**,**e**) are optical microscope images of the top and bottom surfaces, respectively, while (**c**,**f**) are SEM planar view images of the bottom surfaces recorded after a KOH chemical etching. The white marks in (**b**,**c**) highlight the irregularities and explosions at a microscopic scale due to the strong ablation of the surface sample at the glass–air interface, and evidently reduced in the case of micromachining with the adhesive tape (see (**e**,**f**)).

We have made another attempt to optimize the hole quality at the bottom surface of the 200 mm-thick AF32 glass sample by taking advantage of the *Kapton* polyimide adhesive tape. This adhesive tape resists to heat up to 350 °C, and its thickness is about 30 μm. When sticking the tape on the bottom surface of the sample, its refractive index, which is higher than that of the air, causes a reduction of the local intensity of the Bessel beam crossing the interface glass/tape, in contrast to what happens when the Bessel beam crosses the interface glass/air. An example of the BB drilling outcome performed in these conditions and the comparison with a standard hole are shown in Figure 9. The top surface is, as expected, unaffected by the presence of the tape film, while the quality of the aperture at the bottom surface of the glass (observed after the tape removal) is improved with respect to the case of holes drilled without the tape layer. This can be better seen from a comparison of images recorded by means of the scanning electron microscope after a chemical etching of the sample in a 30% KOH solution (Figure 9c,f). In the BB drilling configuration making use of the adhesive layer, we can note that the aperture of the through-hole not only presents more regular features around its border, but it is also characterized by the absence of microcracks, which, on the other hand, are present when no tape is applied under the sample.

### 3.2. PMMA

**Figure 10:**
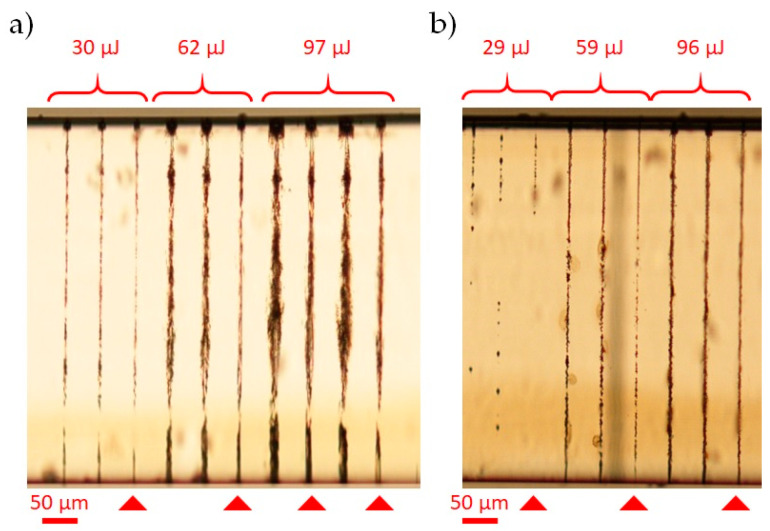
Optical microscope images of microstructures generated in single- (triangles) and four-shots damages in a 500 μm-thick PMMA sample with BB pulse duration of 1 ps (**a**) and 18 ps (**b**) and different energies.

The polymethyl methacrylate (PMMA) is a transparent synthetic resin with very different properties than those of glass (see Table 1). PMMA has a lower coefficient of thermal expansion and much smaller softening point and Young’s modulus, compared to glass. Results of the radiation–matter dynamics during Bessel beam micromachining in PMMA have been reported in [39]. Here, we have studied the interaction of the Bessel beam with a 500 μm-thick PMMA sample, in single and multiple shot regimes, for different pulse energies and for a wide range of pulse durations in the picosecond regime (namely from 1 ps to 18 ps). In the case of multiple shot fabrication, we have considered in particular four laser shots, as these correspond to the number of locally superimposing pulses when using a pulse density of 2000 pulses mm^−^^1^ (our drilling conditions). In Figure 10, we report optical microscope images of microstructures generated in the bulk in single and multiple shot, only for the two extreme values of pulse duration investigated, namely 1 ps and 18 ps, and for different pulse energies. We have verified that, for a given pulse duration, the energy threshold to induce an elongated material modification along the whole sample thickness is typically much lower than that needed in a glass sample of same dimensions. Evident damage inside the PMMA material can be generated with 18 ps pulses and pulse energies still below 100 μJ, highlighting the important role of avalanche ionization (occurring on such time scales) during the nonlinear absorption. In both single and multiple shot regimes, the obtained microstructures are smooth and regular only in a few cases, depending on a careful balancing of pulse energy and pulse duration. In particular, in single shot, continuous traces are visible only for larger pulse durations (as shown in Figure 10b). On the one hand, too strong intensities lead to irregular structures featured by a large internal damage. This is in contrast to typical results obtained in glass [14,24].

**Figure 11:**
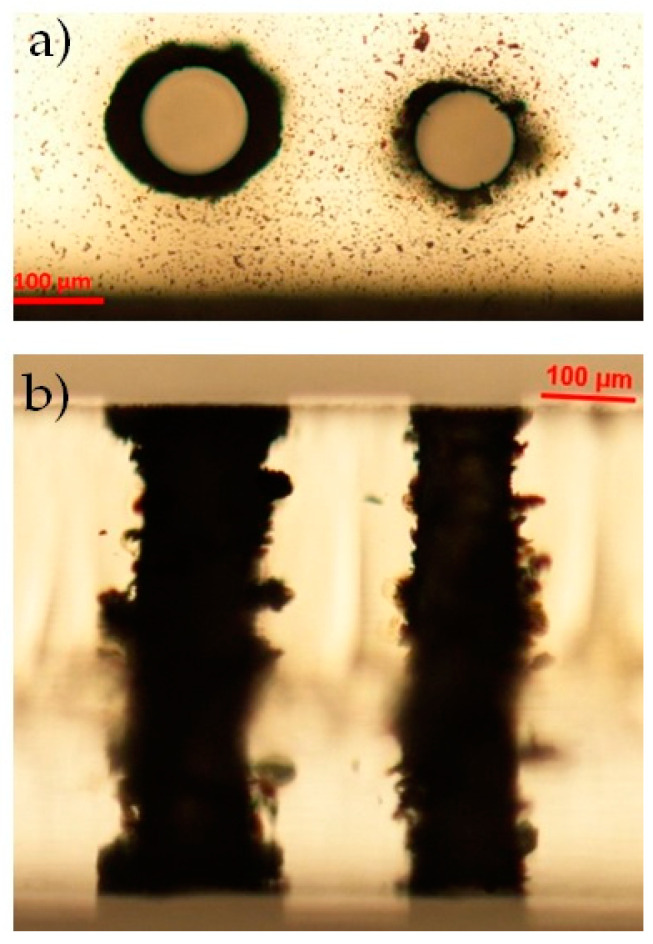
(**a**) Optical microscope images of holes (TOP view) in a 500 μm thick PMMA microfabricated by sending the beam through one of the sample surfaces first and by repeating the drilling process by sending the beam through the other sample surface (bottom surface) after flipping the sample. The left hole is performed with pulse width of 1 ps and a pulse energy of 60 μJ, the right one with 18 ps and a pulse energy of 96 μJ; (**b**) optical microscope images of the lateral view of the through-holes shown in (**a**).

We have applied the BB micro-drilling technique to the PMMA sample, in two configurations, with parameters chosen in accordance with the results obtained in the preliminary radiation–matter interaction study (Figure 10). In particular, for the pulse duration and energy, we have considered those values which have led to the most uniform energy deposition along the sample, for a pulse density of 2000 pulse mm^−1^. We have used in one case a 96 μJ pulse with a duration of 18 ps, and, in a second case, an energy pulse of 60 μJ with a duration of 1 ps. The BB trepanning micromachining process with 100 writing circles was performed three times because of the large sample thickness (exactly as for the Corning glass). No through-apertures could be generated until we repeated the (three-step machining) experiment by flipping the sample upside down. In other words, the drilling process was repeated by injecting the BB onto the PMMA from the other side (what in origin was the bottom surface) by carefully centering the writing trajectories on the previous writing zone. Images of the final results of the top sample surface (after the double 3-passes machining process) and a lateral view of the through-holes are shown in Figure 11: for both sets of experimental parameters, the round apertures are very regular, in contrast to the images of the internal modification, indicating an irregular morphology with a strong and inhomogeneous material modification along the lateral sides. The wider hole is generated when using the 1 ps pulse and 60 μJ pulse energy (thus a higher intensity) in accordance with the results of the behavior of the material modification after multiple shot irradiation. The outcome of the BB drilling process indicates that the laser–matter interaction leads to internal explosions and/or to the melting of the PMMA. We believe that the lower softening point causes a fast PMMA melting and consequently the lower CTE is responsible for a higher expansion of the heated material, with respect to glass, causing a higher pressure in the surrounding cold material. Finally, the very low Young’s modulus allows the deformation of the cold zone even with a low pressure, i.e., with a low increment of temperature. We also remark that the drawback of this experiment was the long fabrication time (double of the time used to drill the Eagle glass of same thickness) due to fact that the 3-step machining process had to be repeated from the reverse side of the sample.

### 3.3. Diamond

**Figure 12:**
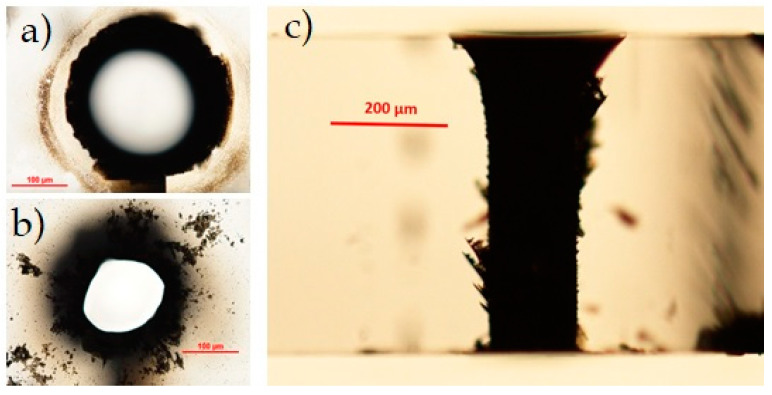
Optical microscope image of a through-hole performed in a 500 μm-thick diamond sample, with 100 writing circles, pulse duration 6 ps and pulse energy 310 μJ. Top (**a**), bottom (**b**) and lateral view (**c**).

In contrast to the dielectric materials considered so far, diamond is composed of carbon atoms arranged in a crystalline structure. It is featured by exceptional properties such as the highest thermal conductivity, high mechanical hardness, wide bandgap, very good optical properties as well as chemical resistance. Except for the CTE, the other parameters (see Table 1) can in fact be orders of magnitude higher compared to glass and PMMA. Laser microstructuring of diamond with Gaussian laser beams was initially reported in the ns or the fs regime [40]. On the other hand, previous bulk micromachining and surface ablation experiments in the picosecond regime with pulsed Bessel beams have already shown the possibility to effectively modify diamond material [41,42]. In particular, the interaction of a Bessel beam propagating along the whole thickness of a diamond sample has led in a multiple-shot regime to graphitized microstructures growing from the bottom surface toward the top surface with length proportional to the number of shots [42]. Progressive damage with ablation of the top diamond surface could also be observed in multiple shot operation. Based on these results, here we have applied the BB drilling technique to a 500 μm synthetic monocrystalline diamond sample. The preliminary results have confirmed the possibility to substantially damage and dig the internal material bulk and consequently to generate through-holes even in diamond. These are shown in Figure 12. In the experiment, the hole was obtained by setting the pulse duration to 6 ps (as for glass and PMMA) and the pulse energy to 310 μJ, using 100 writing circles, and repeating the fabrication process three times. As for the Eagle glass sample, machined with identical parameters, the drilling process lasted 82 min. In contrast to the other dielectrics considered, the size difference between the entrance and exit apertures at the surfaces is much larger. In Figure 12, these apertures are featured by a diameter of about 300 μm and 140 μm, respectively. The bottom surface aperture is not so circular most probably due to a lack of energy, or a misalignment of the Bessel beam inducing some beam astigmatism towards the end of the Bessel zone. Interestingly, the lateral view of the sample under the optical microscope shows a quite regular hole formation across the sample thickness, although little graphitic microstructures may be present close to the walls of the hole [41]. Note that the presence of graphite may affect the BB propagation, as it can induce absorption of portions of the beam especially towards the bottom of the sample, and this may also explain the dimensions unbalance between the top and bottom apertures.

In contrast to the results obtained in glass and PMMA with similar thicknesses, here we do not observe the presence of damage due to shock waves or explosions inside the diamond material. This may be explained by the thermal and mechanical properties of the latter; the fact that diamond has a very high softening point implies that each laser pulse may always interact, during the multiple shot machining, with a cold and strong matter. Moreover, the great Young’s modulus does not allow the deformation of the area surrounding the heat affected zone and the high thermal diffusivity allows fast dispersion of the accumulated heat. We may thus believe that, in diamond shock waves originating from the laser–matter interaction, if they exist, are less strong than those generated during the laser irradiation in the other tested materials. 

## 4. Discussion

In this work, we have applied the Bessel beam hole drilling technique to different dielectric materials with different optical and mechanical properties and different thicknesses ranging from 200 μm to 500 μm. We have successfully demonstrated the possibility to create through-apertures, with diameter size of tens of micrometers, on thick transparent materials, without the need to shift the sample along its thickness (i.e., along the beam propagation direction). This has been achieved by using a Bessel beam with total non-diffracting length in air equal to 570 μm for the 200 μm thick sample or 820 μm for the thicker samples. The microfabrication has been performed by using an amplified low repetition rate high power Ti:Sapphire laser system in the picosecond regime in order to optimize the filamentary deposition of energy inside the materials. We have applied our trepanning-like technique on glass samples with similar properties (Schott AF32 and D263, and Corning Eagle XG borosilicate glasses), on a PMMA sample, and on a synthetic diamond sample. 

For what concerns AF32 glass, we have also tried alternative BB laser micromachining configurations, focusing on the possibility to optimize the hole quality, especially that of the aperture generated on the bottom surface of the sample. The attempts have been made by lowering the difference of the refractive indices at the glass/air interface by means of water and adhesive tape, respectively. The water has been used as the liquid in the liquid assisted laser beam micromachining technique: in this case, the positive and new result has been the generation of high quality through-holes with aspect ratio of about 4:1, with a faster fabrication speed than in air. This has been achieved by starting the trepanning process from the external diameter of the circular writing trajectories, and by using a much smaller number of writing circles than in the standard BB drilling case. This has allowed for reducing the fabrication time by a factor of about three. A second alternative machining configuration in AF32 glass has been performed by taking advantage of the polyimide adhesive tape. The refractive index of the tape is higher than the refractive index of the glass sample, causing a decrease of the local BB intensity at the bottom interface. The quality of the aperture at the top surface was not affected by the presence of the adhesive tape. An improvement of the aperture quality at the bottom surface has been observed, compared to that of the hole generated without adhesive tape. 

The 500 μm-thick PMMA and diamond samples considered in the experiment present different characteristics compared to the glass samples, and have in fact opposite key features not only in terms of hardness (i.e., the softening point temperature), but also and especially for what concerns the Young’s modulus and the thermal diffusivity. We have succeeded in drilling 1:5 aspect ratio through-holes in both these dielectrics but in different conditions, and the results obtained after the laser microfabrication process have revealed that, in these materials, the BB drilling technique leads to different types of bulk modification. In particular, we have shown that it is possible to generate through-holes with a diameter of a few tens of micrometers in thick PMMA, only if repeating the drilling process on the reverse side of the machined sample: moreover, the results of the internal machining are not as satisfactory as those on the sample surface, where the apertures are very regular, and inhomogeneities around the internal walls of the hole are present, unlike the case of glass. In contrast, in the diamond case, despite the presence of graphitization traces on the walls of the hole due to the laser–material interaction, the through-holes appear to be generally regular with a well-defined structure. Counterintuitively, the harder the material, the easier to drill regular holes throughout the bulk. For what concerns the long fabrication times employed in these proof of principle experiments for the generation of through-holes, these will definitely be reduced to the order of seconds or less with a high repetition rate high power laser. The results presented here have proven the possibility to drill through-holes with an innovative technique, in dielectric samples of different types and especially different thicknesses. The advantage of using non-diffracting Bessel beams, already tested for nanochannels or microchannels generation in transparent materials [15,16], is certainly the possibility to have in single shot a radiation–bulk interaction along the whole sample length. It is the plasma column generated in single shot during the nonlinear BB propagation which is responsible for the material modification [43], in contrast to the case of Gaussian beams typically focused down to a localized spot then shifted in depth inside the bulk. Drilling thick materials, with the trepanning-like BB technique, can thus be achieved without any sample translation, allowing for reducing the laser fabrication time, and allowing for changing the relative position between the sample and the beam in two dimensions only, in a geometry that depends on the size of the hole to be obtained. The drawback of this technique may be the request for higher energy than what is usually needed when focusing a Gaussian beam into a well-defined spot, as here the input pulse energy is distributed in the Bessel core along the whole non-diffracting zone. We nevertheless point out that, with current high power and high repetition rate laser sources, this can be easily overcome. While the results shown here are the outcome of proof of principle experiments, future work consists of optimizing the quality of the holes to be drilled depending on the specific applications. These can be, for instance, industrial applications requiring the need of interposer chips, especially based on low CTE materials to be used for through-glass vias technology; microfluidic applications where PMMA is a common material for lab-on-chip devices, or photonics lab-on-chip platforms as those that can be fabricated in diamond [44] to be used for combined microfluidics and biosensing applications.

## Figures and Tables

**Table 1:** Thermal and mechanical specifications of the tested transparent materials. CTE: coefficient of thermal expansion; n: refractive index; Ts: softening point; Y: Young’s modulus (elasticity of the material in tension); α: thermal diffusivity [26,27,28,29,30,31].

Material	Thickness [μm]	CTE [10^−6^K^−1^]	n	T_s_ [°C]	Y [GPa]	α [mm^2^ s^−1^]
AF 32 glass	200	3.2	1.51	717	74.8	N/A
D263 glass	300	7.2	1.52	736	72.9	0.3–0.4
Eagle XG glass	500	3.17	1.5	971	73.6	0.6
PMMA	500	0.5 to 1	1.49	160	3.2	0.11
Diamond	500	1	2.42	4373	1100	1.3 × 10^3^

**Table 2:** Refractive index and cone angles of the Bessel beam in different media.

Medium	Refractive Index	Cone Angle [°]
Air	1	14.7
AF32 glass	1.51	9.7
Water	1.33	11.0
Kapton adhesive tape	1.70	8.6
